# The impact of patient-specific instrumentation on unicompartmental knee arthroplasty: a prospective randomised controlled study

**DOI:** 10.1007/s00167-017-4677-5

**Published:** 2017-08-22

**Authors:** Abtin Alvand, Tanvir Khan, Cathy Jenkins, Jonathan L. Rees, William F. Jackson, Christopher A. F. Dodd, David W. Murray, Andrew J. Price

**Affiliations:** 1 Nuffield Department of Orthopaedics, Rheumatology and Musculoskeletal Sciences, Botnar Research Centre, University of Oxford, Old Road, Oxford, OX3 7LD UK; 2 Nuffield Orthopaedic Centre, Windmill Road, Headington, Oxford, OX3 7LD UK

**Keywords:** Unicompartmental knee arthroplasty, Patient-specific instrumentation, Knee, Arthroplasty

## Abstract

**Purpose:**

Patient-specific instrumentation (PSI) has been proposed as a means of improving surgical accuracy and ease of implantation during technically challenging procedures such as unicompartmental knee arthroplasty (UKA). The purpose of this prospective randomised controlled trial was to compare the accuracy of implantation and functional outcome of mobile-bearing medial UKAs implanted with and without PSI by experienced UKA surgeons.

**Methods:**

Mobile-bearing medial UKAs were implanted in 43 patients using either PSI guides or conventional instrumentation. Intra-operative measurements, meniscal bearing size implanted, and post-operative radiographic analyses were performed to assess component positioning. Functional outcome was determined using the Oxford Knee Score (OKS).

**Results:**

PSI guides could not be used in three cases due to concerns regarding accuracy and registration onto native anatomy, particularly on the tibial side. In general, similar component alignment and positioning was achieved using the two systems (n.s. for coronal/sagittal alignment and tibial coverage). The PSI group had greater tibial slope (*p* = 0.029). The control group had a higher number of optimum size meniscal bearing inserted (95 vs 52%; *p* = 0.001). There were no differences in OKS improvements (n.s).

**Conclusion:**

Component positioning for the two groups was similar for the femur but less accurate on the tibial side using PSI, often with some unnecessarily deep resections of the tibial plateau. Although PSI was comparable to conventional instrumentation based on OKS improvements at 12 months, we continue to use conventional instrumentation for UKA at our institution until further improvements to the PSI guides can be demonstrated.

**Level of evidence:**

Therapeutic, Level I.

## Introduction

The Oxford unicompartmental knee arthroplasty (UKA) is the most commonly performed UKA in England, accounting for 62% of all those performed [8]. Numerous studies have demonstrated excellent long-term survival rates in large cohorts [23, 28, 29]. However, the survival rate of the UKA in joint registries is lower than that achieved in large cohort studies [8]. Registry data have highlighted the role of experience and surgical caseload in determining successful outcomes after UKA, demonstrating better results for UKAs performed by high-volume surgeons in high-volume centres [2, 21]. Technical difficulty of UKA is thought to contribute significantly to the variations observed in surgical performance and results [9, 10, 14, 22, 23, 30, 32, 35]. The implication is that those aiming to undertake UKA must be adequately trained and perform a certain number of cases annually. In addition, particularly for inexperienced and low-volume surgeons, there is a need for technological innovations to improve surgical accuracy. A technological tool that has recently received significant attention is patient-specific instrumentation (PSI) for knee arthroplasty [1, 20, 33]. This uses 3-D imaging techniques (MRI or CT) and rapid prototyping technology to produce patient-specific guides for making the femoral and tibial bone resections. PSI systems aim to improve accuracy of implant positioning in addition to the reduction of fat embolism risk, instrument inventory, and operative time [3, 7, 25, 31, 37]. The PSI system developed for the Oxford UKA is the “Signature” system (Zimmer Biomet Inc, Warsaw, IN, USA). It aims to simplify and improve the accuracy of surgical implantation, which is important in determining the outcome of the procedure [5, 13]. If this PSI system works reliably, it is likely to be particularly useful for low-volume UKA surgeons. However, before this can be done, experienced surgeons should assess the system’s reliability. Recent case-series and laboratory-based experiments have demonstrated that PSI technology can improve component positioning during UKA surgery [11, 15, 18, 36]. However, the effect of this technology has received little attention in randomised studies.

Before inexperienced surgeons use such technology, experienced UKA surgeons must evaluate its safety and reliability in order to ensure that no harm comes to patients. The purpose of this prospective randomised controlled trial (RCT) was therefore to compare the accuracy of implantation and functional outcome of mobile-bearing medial UKAs implanted with and without PSI by experienced UKA surgeons.

## Materials and methods

This single-centre parallel-design RCT was conducted between 2012 and 2014. Ethical approval was obtained, and the trial was registered with the United Kingdom National Research Ethics Service committee (REC reference: 11/H0605/1**)** and the hospital review board. The study was registered at ClinicalTrials.gov (NCT02748096). Four expert OUKA surgeons (DWM, CAFD, AJP, and WFMJ) performed all of the procedures in this study. These surgeons had previously performed a total of ten Oxford UKA procedures using this PSI system and so were familiar with the technique. Patients who were being placed on the waiting list for a medial OUKA and met the entry criteria for the trial, were asked whether they would be willing to receive further information about participation in the study. They were provided with a study information leaflet that they could read in their own time. A member of the research team (AA) subsequently contacted the patients in order to determine whether they would agree to take part in the study and enrolled them onto the study.

Inclusion criteria were standard for medial OUKA:

- Both cruciate ligaments functionally intact.
- Full-thickness cartilage in the lateral compartment.
- Correctable intra-articular varus deformity (based on clinical assessment).
- Full-thickness cartilage loss in the medial compartment.

Exclusion criteria were as follows:

- Contra-indication for MRI.
- All forms of inflammatory arthritis.

**Fig. 1:**
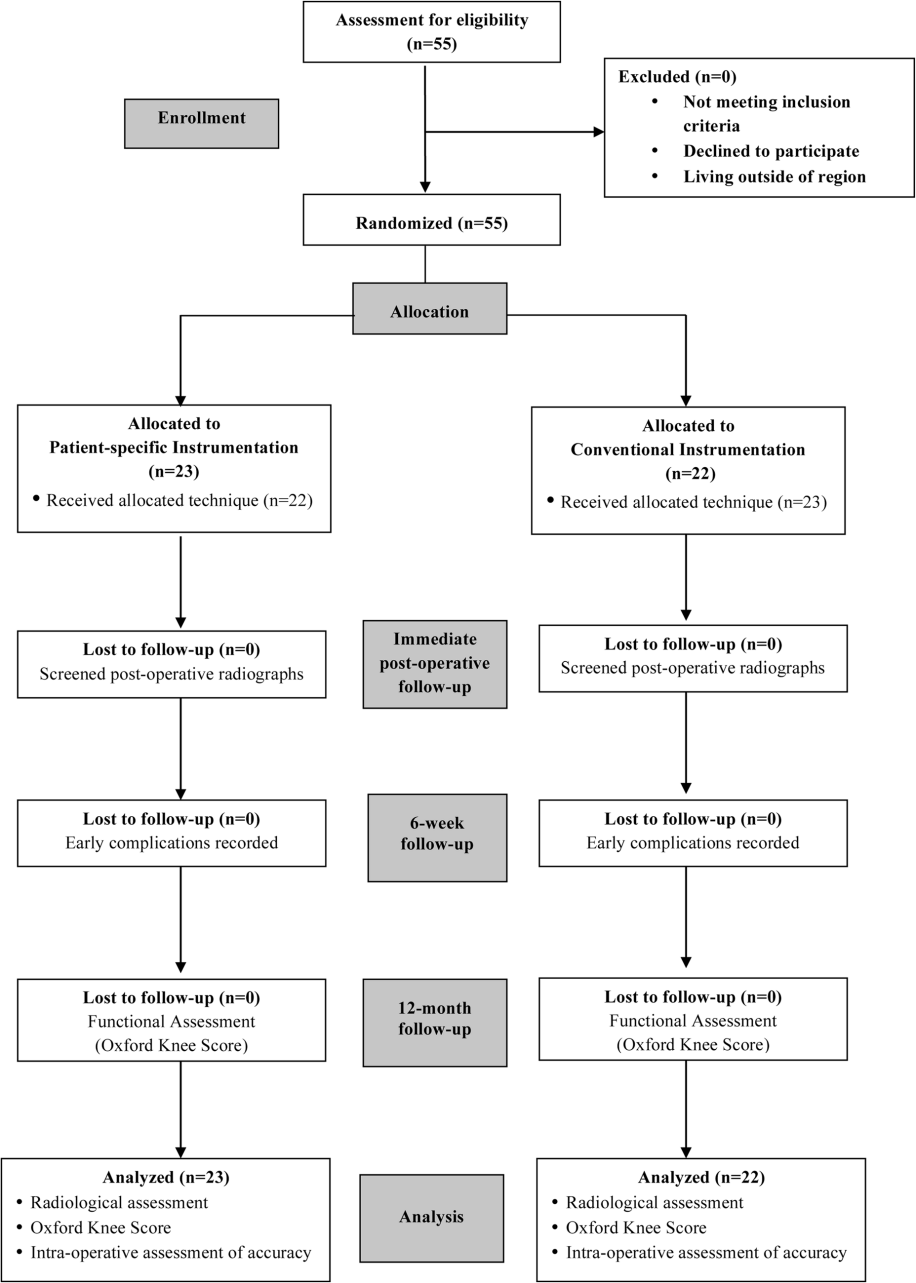
A consolidated standards of reporting trials (CONSORT) diagram showing the flow of patients in the study

The flow of patients through the trial is presented in Fig. 1. Patients were randomised to either PSI or Conventional Instrumentation (CI) group by one of the investigators (AA). The Oxford Microplasty instrumentation was used to implant all UKAs in the CI group. There were 23 patients (23 knees) in the PSI group and 22 patients (22 knees) in the CI group. Randomisation was performed using sealed opaque envelopes. Blinding of the operating surgeons and patients was not possible owing to the surgeon needing to confirm the PSI plans, and the patients undergoing pre-operative MRI scans. PSI group patients underwent an MRI scan using the protocol outlined by the PSI manufacturers to plan development of the PSI guides. The preliminary plan indicating prosthesis size, positioning, alignment, and proposed bone resection levels was reviewed by the surgeons who accepted the default pre-operative plans unless gross errors were present. The patient-specific cutting guides were then manufactured and sent for sterilisation.

### Operative technique

All patients received a mobile-bearing medial OUKA via a minimally invasive approach and high thigh tourniquet. Intra-operatively, the bone cuts were made through the PSI guides without the use of any intra- or extramedullary instrumentation on the femoral side but with a tibial extramedullary guide for some cases. The subsequent milling process and all soft tissue balancing were performed manually in the standard fashion. In cases where the surgeon felt that the PSI guides did not fit appropriately, the conventional instrumentation was utilised. This situation occurred during three cases. Post-operatively, the need to have a blood transfusion, and the change haemoglobin levels were also recorded. Oxygen saturation levels over the first 24 h post-surgery were also recorded. Screened anteroposterior (AP) and lateral (LA) post-operative radiographs were performed prior to discharge. Patients attended the physiotherapy ward discharge clinic at 6 weeks. A further clinical review was performed at 12 months at which point Oxford Knee Scores (OKS) were recorded.

### Outcome measures

#### Primary outcome measure: radiological assessment of component positioning

This was performed on screened post-operative radiographs, according to the parameters proposed by developers of the OUKA [12]. These describe optimum ranges for alignment and fit of the components (Table 1). These parameters were measured by two independent (blinded) assessors (AA and TK) using custom software developed in Matlab (v.7.0, The MathsWork Inc., MA, USA). The radiographs were converted from DICOM to JPEG files before analysis. For measurements of distance in millimetres, the software used a conversion factor calculated by dividing the known diameter of the femoral component (provided by the manufacturer) by the diameter of a circle fitted to the component in pixels. For the prosthesis component alignment measurements (i.e. angles), the software uses points on the diaphysis and metaphysis of the femur and tibia to map out the vertical axes of the tibia and femur. Selecting additional points on the femoral and tibial components of the OUKA enables the software to calculate the sagittal and coronal alignment of the prosthesis. These radiographic parameters were measured twice on two separate days, in order to determine inter-observer and test–retest reliability. The intra-class correlation coefficient (ICC) for all the radiographic measurements undertaken using this system was excellent with all measured parameters having an ICC >0.8.

**Table 1:** Optimum ranges for the radiographic parameters used to assess implant positioning and alignment

Radiographic parameter	Optimum position
Femoral component: varus/valgus angle (AP radiograph)	<10.0° varus to <10.0° valgus
Femoral component: flexion/extension angle (LA radiograph)	15.0° flexion to <0° extension
Tibial component: varus/valgus angle (AP radiograph)	<5.0° varus to <5.0° valgus
Tibial component: posterior tilt (slope) angle (LA radiograph)	Within ±5.0° of the 0° baseline (“Baseline” taken as 7.0° posterior tilt but this is recorded as 0°)
Tibial component: medial fit (AP radiograph)	Flush or <2.0 mm overhang
Tibial component: anterior fit (LA radiograph)	Flush or <2.0 mm overhang
Tibial component: posterior fit (LA radiograph)	Flush or <5.0 mm underhang

### Secondary outcome measures

#### Assessment of functional outcome

This was determined using the pre-operative and 12-month follow-up OKS (with the maximum possible score of 48). The OKS is a validated 12-item questionnaire that addresses pain and functional disability in relation to knee problems [24].

The remaining outcome measures were used to further evaluate the efficiency and safety of the PSI system.

#### Intra-operative assessment of surgical accuracy

- Correspondence between the *implanted* and *planned* component sizes.
- The need to perform a horizontal tibial “re-cut”.
- Tracking of the meniscal bearing (measured as the distance from bearing to the metal upright of the tibial component in flexion and extension).
- Size of the meniscal bearing inserted (optimum bearing size considered as 3 or 4 mm).

### Statistical analysis

The sample size was calculated from a previous study that used similar radiological assessments to compare OUKAs performed using conventional instrumentation, with those performed using computer navigation [16]. In this study, the standard deviation of the tibia varus/valgus angle for the control group was 3.6°. Assuming a minimum clinically important difference of 3°, the standard mean difference would be 0.8. Hence with a power of 0.8 and significance level of 0.05, a total sample size of 44 patients (22 in each group) was required.

The Shapiro–Wilk test showed that all data, except “bearing alignment”, were normally distributed. Parametric tests were applied to normally distributed data. The independent group *t* test was used to compare group demographics, radiographic parameters, OKS, and operative time. The Pearson Chi-square test was used to compare “radiographic parameter outliers” and “bearing sizes”. The Mann–Whitney *U* test was used to compare “bearing alignment”. The inter-observer and test–retest reliability of the radiographic parameters was assessed using the ICC. Statistical analyses were performed with IBM SPSS Statistics version 22 (Chicago, IL, USA). A *p* value <0.05 was considered statistically significant.

## Results

### Participant demographics

The groups were evenly matched in terms of age, sex, American Society of Anesthesiologists (ASA) grade, and body mass index (BMI) (Table 2).

**Table 2:** Summary of the study subjects’ demographics and operative time

Demographic	Patient-specific instrumentation (*n* = 23)	Conventional instrumentation (*n* = 22)	*p* value
Mean age (range) in years	66.9 (52.2–77.1)	68.2 (51.0–88.2)	n.s.
Sex (M:F)	10:13	13:9	n.s.
Median ASA (range)	2 (1–3)	2 (1–3)	n.s.
Mean body mass index (range)	29.8 (23.8–40.3)	31.8 (22.2–39.5)	n.s.
Mean operative time (range) in minutes	75.3 (53.0–90.0)	63.5 min (50.0–82.0)	0.001**

*ASA* American Society of Anaesthesiologists grade

** Statistically significant difference

### Primary outcome measure: radiological assessment of component positioning

No patients were lost to follow-up. The ICCs for all radiographic parameters were greater than 0.80 indicating high test–retest and inter-observer reliability. The mean radiographic parameter values are presented in Table 3. There were no statistical differences in component positioning between the groups, except for the “posterior tilt” of the tibial component. The proportion of cases that were positioned outside the optimum ranges (outliers) are summarised in Table 4. There was no significant difference between the numbers of outliers in the two groups.

**Table 3:** Summary of the radiographic parameters in the patient-specific instrumentation and conventional instrumentation groups

Radiographic parameter	Patient-specific instrumentation	Conventional instrumentation	*p* value (means)
	Mean (SD)	Mean (SD)	
Femur: varus/valgus angle	0.9° varus (4.0)	1.8° varus (3.0)	n.s.
Femur: flexion/extension angle	9.1° flexion (3.0)	8.8° flexion (4.8)	n.s.
Tibia: varus/valgus angle	3.5° varus (2.9)	4.0° varus (2.1)	n.s.
Tibia: posterior tilt angle	1.8° superior (2.8)	3.7° superior (2.1)	0.029**
Tibia: medial fit	0 mm (1.0)	1.0 mm underhang (1.3)	n.s.
Tibia: anterior fit	1.0 mm underhang (0.9)	1.0 mm underhang (0.7)	n.s.
Tibia: posterior fit	0 mm (0.8)	0 mm (0.9)	n.s.

*SD* standard deviation

** Statistically significant difference

**Table 4:** Proportion of radiographic parameter outliers in the patient-specific instrumentation and conventional instrumentation groups

Radiographic parameter	Patient-specific instrumentation (*n* = 23)	Conventional instrumentation (*n* = 22)	*p* value
	Number of outliers [%]	Number of outliers [%]	
Femur: varus/valgus angle	1 (varus) [4%]	None	n.s.
Femur: flexion/extension angle	None	1 (flexed) [5%]	n.s.
Tibia: varus/valgus angle	5 (all varus) [22%]	6 (all varus) [27%]	n.s.
Tibia: posterior tilt angle	2 (1 superior, 1 inferior) [9%]	5 (all superior) [3%]	n.s.
Tibia: medial fit	None	1 (underhang) [5%]	n.s.
Tibia: anterior fit	None	None	NA
Tibia: posterior fit	None	1 (overhang) [5%]	n.s.

Comments in brackets indicate the direction of the outliers

### Secondary outcome measures

#### Assessment of functional outcome

Table 5 summarises the mean OKS results for the two groups. There was no significant difference in the mean OKS improvements between the two groups.

**Table 5:** Pre-operative and 1-year Oxford Knee Score results of the two groups

Mean Oxford Knee Score, OKS (range)	Patient-specific instrumentation (*n* = 23)	Conventional instrumentation (*n* = 22)	*p* value
Pre-op OKS	24.1 (12–38)	23.3 (10–37)	n.s.
Post-op OKS	42.4 (21–48)	41.5 (26–48)	n.s.
Δ OKS	18.3 (4–31)	18.2 (5–31)	n.s.

#### Intra-operative assessment of surgical accuracy

The PSI guides could not be used for 3 (13%) patients, as they would not fit onto the patient’s native anatomy. This occurred for both the femoral and tibial guides in one patient, and the tibial guides only for two patients. With regard to the planning of implant sizes, 21 (91%) of the femoral components implanted corresponded to the sizes that were planned pre-operatively. On the other hand, only 11 (48%) of the tibial components implanted corresponded to the sizes that were planned pre-operatively.

Table 6 summarises the cases in each group that required a tibial plateau “re-cut” and the results for tracking of the meniscal bearing.

**Table 6:** Proportion of cases in each group that required a tibial plateau “re-cut” and the results for tracking of the meniscal bearing in flexion and extension

	Patient-specific instrumentation (*n* = 23)	Conventional instrumentation (*n* = 22)	*p* value
Cases requiring horizontal “re-cut” of the tibial plateau	3	2	n.s.
Median (and interquartile range) distance between the bearing to the metal upright of the tibial component wall	Flexion: 1 mm (1–2) Extension: 2 mm (1–2.5)	Flexion: 1 mm (1–1.5) Extension: 1 mm (1–2)	n.s. n.s.

#### Bearing size implanted

**Fig. 2:**
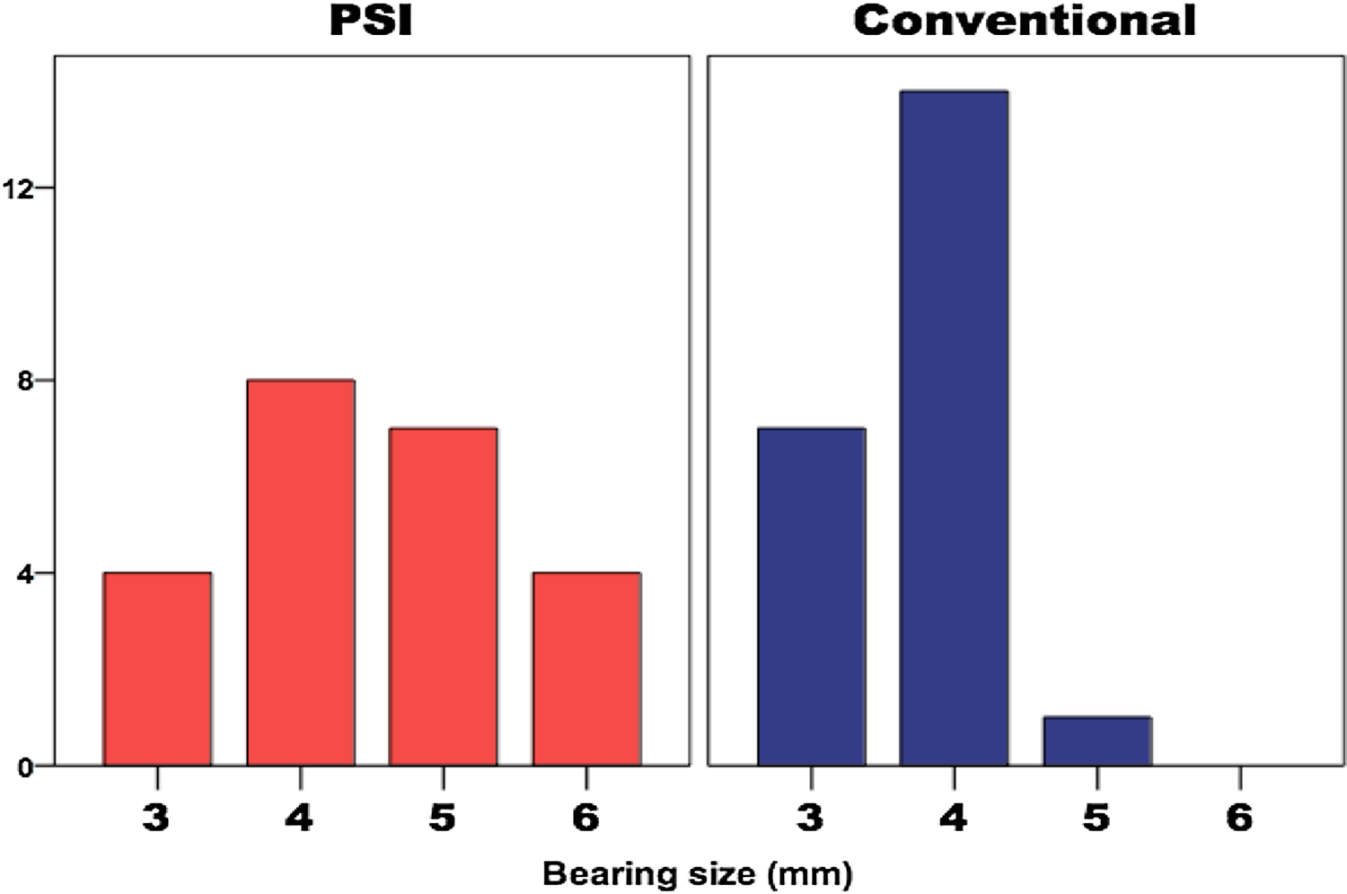
Bar chart demonstrating the differing bearing sizes implanted in each group

All but one of the CI group had a size 3 or 4 mm (i.e. optimum size) meniscal bearing inserted compared with only 12 in the PSI group (Fig. 2). This difference was statistically significant (*p* = 0.001).

#### Complications

There were two superficial wound infections (one in each group), which were treated by the patients’ family practitioners with oral antibiotics. At the 1-year review, no further complications had occurred and no prostheses had been revised.

## Discussion

The most important finding of this study was that the OUKA PSI system had similar accuracy to conventional instrumentation with regard to femoral component positioning and alignment, but was less accurate for tibial component positioning resulting in unnecessarily deep tibial resections. Although there was no difference in functional outcome between the two cohorts at one year, we conclude that, in the hands of experienced UKA surgeons, the iteration of the PSI system assessed in the current study was not better than current (conventional) instrumentation.

An important finding, which suggests that the PSI was inferior to the conventional system, was that a significantly lower number of cases in the PSI group had the “optimum” bearing size implanted (3 or 4 mm; 52% of the PSI group compared to 95% of the CI group). The PSI group tended to have thicker bearings, which are associated with higher failure rates in the long term [28]. In addition, we believe that deep resections pose the danger of damaging the deep fibres of the medial collateral ligament and the bone may be weakened with an increase in the risk of pain and fracture. Since this study was undertaken, the PSI algorithm has been altered so that the resections are not so deep. Another important finding was that in some cases, the tibial PSI guides did not register adequately on the native tibia. Thus, further work is needed to improve the tibial PSI guides. The unexpected finding of an increased operative time observed in the PSI group could most likely be explained by the fact that the surgeons were intentionally devoting some extra time to evaluating the PSI guides intra-operatively so that they could provide meaningful feedback to the PSI development engineers.

The radiological assessment of component alignment and positioning was based on the recommendations of the OUKA design manual and the literature [6, 13]. Although no difference was found in the number of outliers between the PSI and CI groups, the use of PSI resulted in improvements in optimum posterior tibial slope and the optimum medial fit of the tibial component. The clinical relevance of these minor improvements is not clear. In contrast to the current study, a laboratory-based study demonstrated that PSI was more accurate than CI and was equivalent to a robot-assisted system [15] and a case-series by Volpi et al. [36]. showed that PSI was “highly accurate in reproducing what the surgeon had planned”. However, more recent studies have not been able to replicate these promising early results. A retrospective series of 30 Oxford “Signature” UKAs demonstrated similar radiological outcomes as the present study, and comparable results to standard instrumentation [17]. The authors also reported difficulties with use of the tibial guides. Furthermore, a recent study utilising CT scans to determine the accuracy of component positioning and alignment using the OUKA PSI system found that there was no agreement between the pre-operative plans and the post-operative component alignment for the femoral component angle in the sagittal and axial plane and for the tibial component angle in the coronal plane [34]. Similarly, recent RCTs comparing PSI guides with conventional instrumentation for implanting TKAs have demonstrated either no additional benefit [19, 38] or detrimental effects on implant positioning when using PSI [27].

With regard to functional outcome, we found no significant difference in OKS improvement. There is little previous work regarding the effect of PSI on functional outcome, but a case-series by Bell et al. [4] which assessed a different UKA PSI system mirrored our results and demonstrated excellent functional outcome (OKS and Forgotten Joint score) at one year. Only one randomised study has evaluated functional outcome following in UKA, using a different PSI system by using 3-D gait analysis, the SF-12, and the Knee Society Score [26]. This study demonstrated no significant differences at one year between the two groups.

This study had some limitations. Firstly, the surgeons performing the cases were highly experienced OUKA surgeons with extensive previous experience of the CI. These experts are therefore likely to have a very low number (if any) of surgical outliers when using the CI. This potential bias is further amplified by the surgical learning curve associated with the use of the PSI. A concerted effort was made by the surgeons to address this issue by familiarising themselves with the PSI in a previous pilot study. Nevertheless, the previous experience of the surgeons with the conventional instrumentation is very likely to have favoured the CI group and hence it would have been difficult to demonstrate more superior surgical accuracy using PSI. Secondly, the radiological assessment was based on coronal and sagittal alignment. Further evaluation of component rotation using CT scans would have been desirable but was limited by resources. Nevertheless, the radiographic parameters were those recommended by the designers of the OUKA and previous studies have validated their use [6, 13]. The current study was powered to detect important differences in component alignment and positioning. It is likely to have been underpowered for detecting clinically important changes in OKS. However, the rationale for the current study can be justified based on patient safety factors. Finally, the current study did not evaluate the long-term implant survival and risk of revision surgery—a factor that which is key in determining the efficacy of PSI technology. PSI technology is an exciting development that has received significant attention over the past decade. However, further evaluation and improvement of the PSI guides used in the current study are necessary before they can be utilised on a regular basis in day-to-day clinical work.

## Conclusion

The results of this study demonstrate that high-volume OUKA surgeons can achieve similar results, in terms of component alignment and functional outcome when using PSI guides for OUKA. However, intra-operative assessment of the tibial guides by expert OUKA surgeons suggests that although the early “Signature” PSI system design used for this study can safely be used by experienced surgeons, it is not appropriate for inexperienced surgeons.
